# Transposons, environmental changes, and heritable induced phenotypic variability

**DOI:** 10.1007/s00412-014-0464-y

**Published:** 2014-04-22

**Authors:** Lucia Piacentini, Laura Fanti, Valeria Specchia, Maria Pia Bozzetti, Maria Berloco, Gino Palumbo, Sergio Pimpinelli

**Affiliations:** 1 Istituto Pasteur, Fondazione Cenci-Bolognetti and Dipartimento Di Biologia e Biotecnologie, Università di Roma “La Sapienza”, Piazzale Aldo Moro 5, 00185 Rome, Italy; 2 Dipartimento di Scienze e Tecnologie Biologiche ed Ambientali (DiSTeBA), University of Salento, Lecce, Italy; 3 Dipartimento di Biologia, Università degli Studi di Bari Aldo Moro, 70121 Bari, Italy

**Keywords:** Stress, Hsp90, Transposons, Evolution

## Abstract

The mechanisms of biological evolution have always been, and still are, the subject of intense debate and modeling. One of the main problems is how the genetic variability is produced and maintained in order to make the organisms adaptable to environmental changes and therefore capable of evolving. In recent years, it has been reported that, in flies and plants, mutations in *Hsp90* gene are capable to induce, with a low frequency, many different developmental abnormalities depending on the genetic backgrounds. This has suggested that the reduction of Hsp90 amount makes different development pathways more sensitive to hidden genetic variability. This suggestion revitalized a classical debate around the original Waddington hypothesis of canalization and genetic assimilation making Hsp90 the prototype of morphological capacitor. Other data have also suggested a different mechanism that revitalizes another classic debate about the response of genome to physiological and environmental stress put forward by Barbara McClintock. That data demonstrated that Hsp90 is involved in repression of transposon activity by playing a significant role in piwi-interacting RNA (piRNAs)-dependent RNA interference (RNAi) silencing. The important implication is that the fixed phenotypic abnormalities observed in *Hsp90* mutants are probably related to de novo induced mutations by transposon activation. In this case, Hsp90 could be considered as a mutator. In the present theoretical paper, we discuss several possible implications about environmental stress, transposon, and evolution offering also a support to the concept of evolvability.

Which is more changeable: reality or how we explain it?

(The authors)

## Heritable stress-induced phenotypic variability

After Darwin’s book on the origin of species by natural selection, the theory espoused by his predecessor Lamarck (1809) was never completely abandoned. Over time, the observation of certain natural phenomena has occasionally resurrected the concept of the heredity of acquired characters (see Koonin and Wolf 2009 for a discussion). One of the most striking cases of apparently heritable acquired characters was observed by Conrad Waddington, who observed that some phenotypic traits, induced in *Drosophila* pupae by heat shock treatment and then selected for a number of generations in the presence of heat shock, became heritable, seemingly showing that induced phenotypic traits could be inherited through the germ line. To provide a Darwinian explanation of these results, Waddington elaborated the concepts of “canalization and assimilation” (Waddington 1942, 1959). Waddington hypothesized the existence of a preexisting cryptic genetic variation that remains hidden due to robustness in the developmental process which he called “canalization” (Waddington 1942). If an environmental stress is strong enough to overcome this robustness, the developmental pathway can change through the expression of a cryptic genetic variant. This variant can then be selected and become heritable by an “assimilation” process (Waddington 1959).

During the last few years, two main molecular explanations, perhaps complementary rather than alternative, for Waddington’s observation have been proposed. In flies and plants, when the activity of Hsp90 is reduced via silencing from mutations or treatment with specific inhibitors such as geldanamycin, a wide spectrum of phenotypic variants is induced (Rutherford and Lindquist 1998; Queitsch et al. 2002). Under selection, these variants can occasionally be fixed and stably transmitted even if Hsp90 function is restored in subsequent generations. Other recent observations seem to support the existence of such a mechanism in the cavefish *Astyanax mexicanus* (Rohner et al. 2013). The interpretation was that Hsp90 is a capacitor of morphological evolution: It buffers a preexisting genetic variation that is not expressed and accumulates under neutral conditions; its inhibition will induce the expression of this variation. The stress-sensitive storage and release of genetic variation by Hsp90 would aid adaptive evolution. Recent data have suggested other genetic and epigenetic mechanisms that could also be involved in the storage of genetic variation (Burga et al. 2011; Gangaraju et al. 2011). Moreover, it has been shown that also epigenetic variation could be transgenerationally inherited (Sollars et al. 2003; Tariq et al. 2009).

**Fig. 1:**
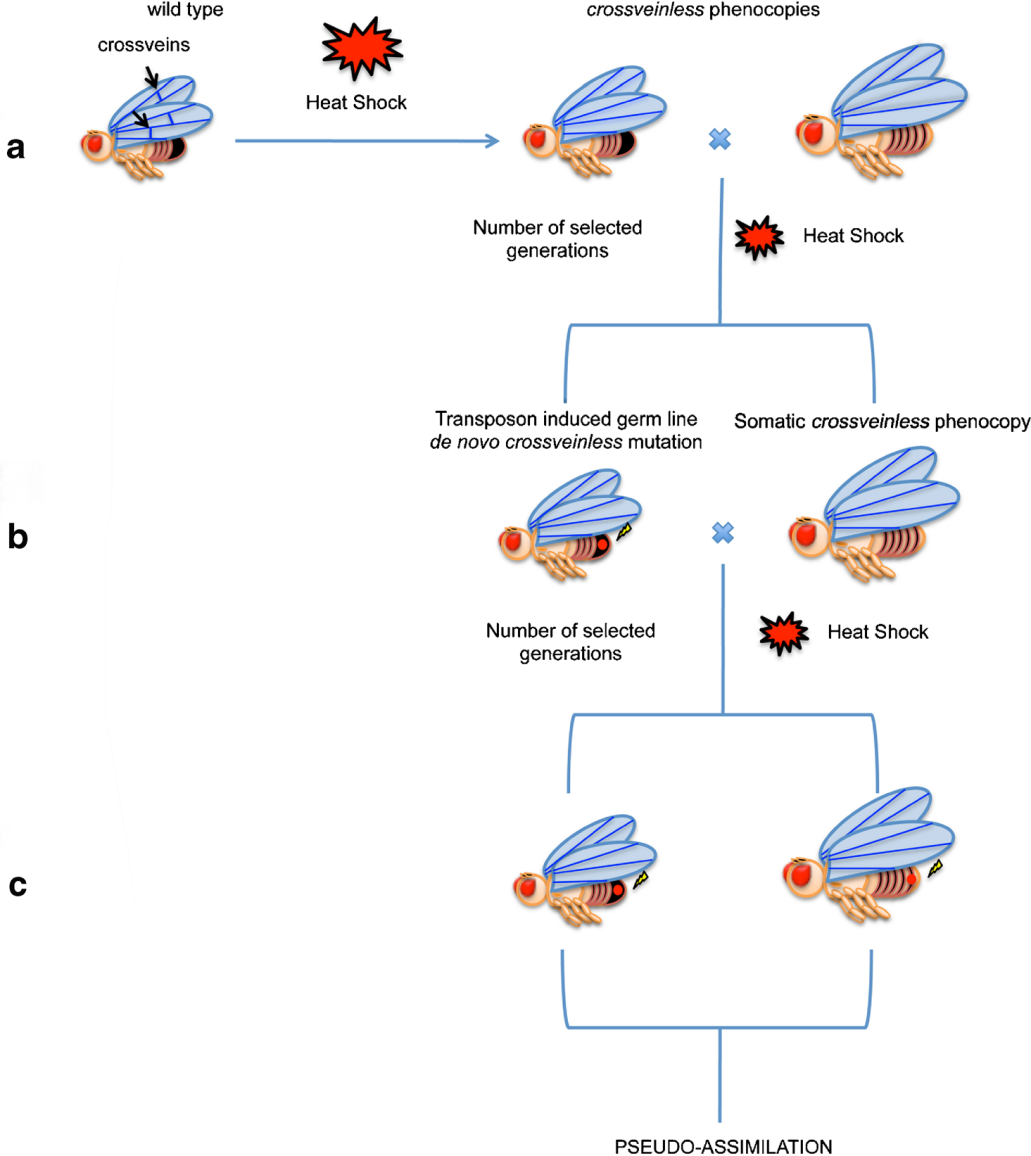
Assimilation of a stress-induced phenotypic trait. **a** Morphological change (phenocopy) induced by stress. **b** A mutation producing the same phenotype can be induced by the activity of transposons in the germ line of the same individual or other individual. **c** Co-selection of the somatically-induced morphological change with a corresponding germ line mutation eventually make the phenocopy apparently heritable (assimilation)

Another study performed in *Drosophila* (Specchia et al. 2010) has suggested a different genetic mechanism that could contribute to stress-induced phenotypical variability. It has been demonstrated that Hsp90 regulates silencing mechanisms mediated by piwi-interacting RNAs (piRNAs), a class of germ line-specific small interfering RNAs (siRNAs) known to maintain repetitive sequences and transposons in a repressed state (Ghildiyal and Zamore 2009). Functional alteration of Hsp90 results in transposon activation in the germ cells and the induction of a wide range of phenotypic variants. Notably, molecular analysis of a phenotypic variant isolated in an Hsp90 mutant strain showed a transposon insertion in the corresponding gene. In addition, other mutations that impair piRNA biogenesis were capable of inducing phenotypic variation, further indicating that the expression of morphological variation is related to the disruption of the piRNA silencing mechanism. On the basis of these observations, it has been suggested that Hsp90 could be a suppressor of transposition of mobile elements. Molecular bases of Hsp90’s role in the piRNA-mediated transposable element (TE) silencing have also been demonstrated (Gangaraju et al. 2011; Olivieri et al. 2012; Izumi et al. 2013). Thus, the absence of the product of Hsp90 promotes mutation-activating transposable elements through impairment of RNA interference (RNAi) silencing. In this scenario, Hsp90 could be seen as a mutator, and Waddington’s results could be at least partially explained as follows: Stress alters developmental processes and produces phenotypic variants. The induction of transposon activity may generate germ line mutations producing the same phenotypic variants (see also Sato and Siomi 2010 for a discussion of these models). Selection, under stress, in following generations of a somatically induced phenotypic variant may also allow the co-selection of a corresponding germ line mutation thus making it apparently heritable such as phenotypic variant (Fig. 1). A support to this model comes also from previous data showing that heat shock treatment induces transposition of mobile elements (Ratner et al. 1992).

Although all the above-mentioned models are surely very interesting for their evolutionary implications, here, we wish to specifically discuss the relationship between environmental stress and transposons and its relevance in evolutionary processes in a Darwinian landscape.

## Environmental stress and transposon activity

“We should always believe our observations, however bizarre they may seem.

Maybe they’re trying to tell us something.”

Barbara McClintock

Barbara McClintock, in her science article on “The significance of responses of the genome to challenge,” reported examples of genomic reactions to stress by activation of mobile elements and, with her *visionary mind*, suggested that the resulting restructured genomes may underlie the formation of new species (McClintock 1984). Since then, a plethora of papers proposing the causal involvement of transposons in evolutionary processes has been continuously published (see Huang et al. 2012; Lisch 2013; Gbadegesin 2012 for reviews). Several examples of transposons as evolutionary tools have been described, and transposons have been recognized as important contributors to reshaping genomes throughout evolution by creating new regulatory elements, gene mutations, and chromosome rearrangements such as duplications, inversions, and translocations that permit adaptation (Kidwell and Lisch 2001; Kazazian 2004). Recently, various reviews and theoretical works have been published which propose a basic hypothesis: Drastic environmental change can disrupt the mechanism of transposon silencing and induce an explosion of transposon activity with the creation of genetic variability. The consequent burst of transpositions could help macroevolutionary processes (Zeh et al. 2008; Oliver and Greene 2009; Oliver and Greene 2011; Rebollo et al. 2010). This hypothesis, however, has its problems: What little experimental evidence that there is so far shows that, in organisms under stress, there is no direct correlation between the increase in transposon transcripts and the rate of transposition. For example, what is generally observed in plants is a large accumulation of transposon transcripts accompanied by a moderate increase in the rate of transposition and in some cases even no increase (see Ito 2013 for discussion). In particular, it has been shown that in plants, heat shock, as well as other biotic and abiotic stresses, induces massive transposon-transcript accumulation (Zeller et al. 2009; Tittel-Elmer et al. 2010), and in mammals, the stress activates short interspersed element (SINE) expression (Li et al. 1999; Liu et al. 1995). It is possible to speculate that, while a low rate of transposition is efficient in producing genetic variability, an accumulation of transposon transcripts plays some regulative role in genome activity. It was recently discovered that transposable elements can regulate non-neighboring genes through the transacting nature of small interfering RNAs. Two reports, from *Drosophila* and *Arabidopsis*, have in fact demonstrated that epigenetically and developmentally regulated bursts of TE expression produce gene-regulating small RNAs (Rouget et al. 2010; McCue et al. 2012; see also Wheeler 2013 for review). Based on these examples, it is reasonable to think that, through small RNAs, transposon transcripts could widely influence the genome transcriptome. Furthermore, it was also shown that an increase of the human Alu RNA after exposure of the cells to different types of stress regulates protein synthesis by maintaining a translational homeostasis (Chu et al. 1998).

Perhaps, under stress, both the mechanisms of transposon silencing and those of transposition are completely or partially inactivated. The consequence would then be a substantial accumulation of transcripts but a low efficiency of transposition. As we will discuss later, the low efficiency of the insertion mechanisms that could be considered a reaction norm describes the susceptibility of an organism in reacting to environmental stimuli. In other words, the absence of massive transposition is probably because a moderate strength of the reaction norm had previously been selected for. This is quite logical since massive transposition would be lethal in individuals with strong susceptibility, who would thus be eliminated.

That massive transposition that causes drastic deleterious effects is demonstrated by the well-known phenomenon of hybrid dysgenesis in *Drosophila* (Bingham et al. 1982; Bucheton 1990). In some cases, crosses between individuals from natural populations with individuals from laboratory stocks produce sterile hybrid females. The sterility is due to embryo lethality caused by an extensive transposition of mobile elements.

Thus, bursts of intense transposition, if triggered by particularly drastic environmental stresses, would have been involved mostly in causing the extinction of species rather than in the speciation process. However, they may have also played a special role in speciation as physiological isolating mechanisms, causing a sort of hybrid dysgenesis in crosses between incipient species.

Since Hsp90 is one of the factors involved in the reaction to stress, it is reasonable to think that stress activates transposons by affecting piRNA biogenesis through Hsp90, thus establishing a possible causal correlation between stress and transposon activity.

**Fig. 2:**
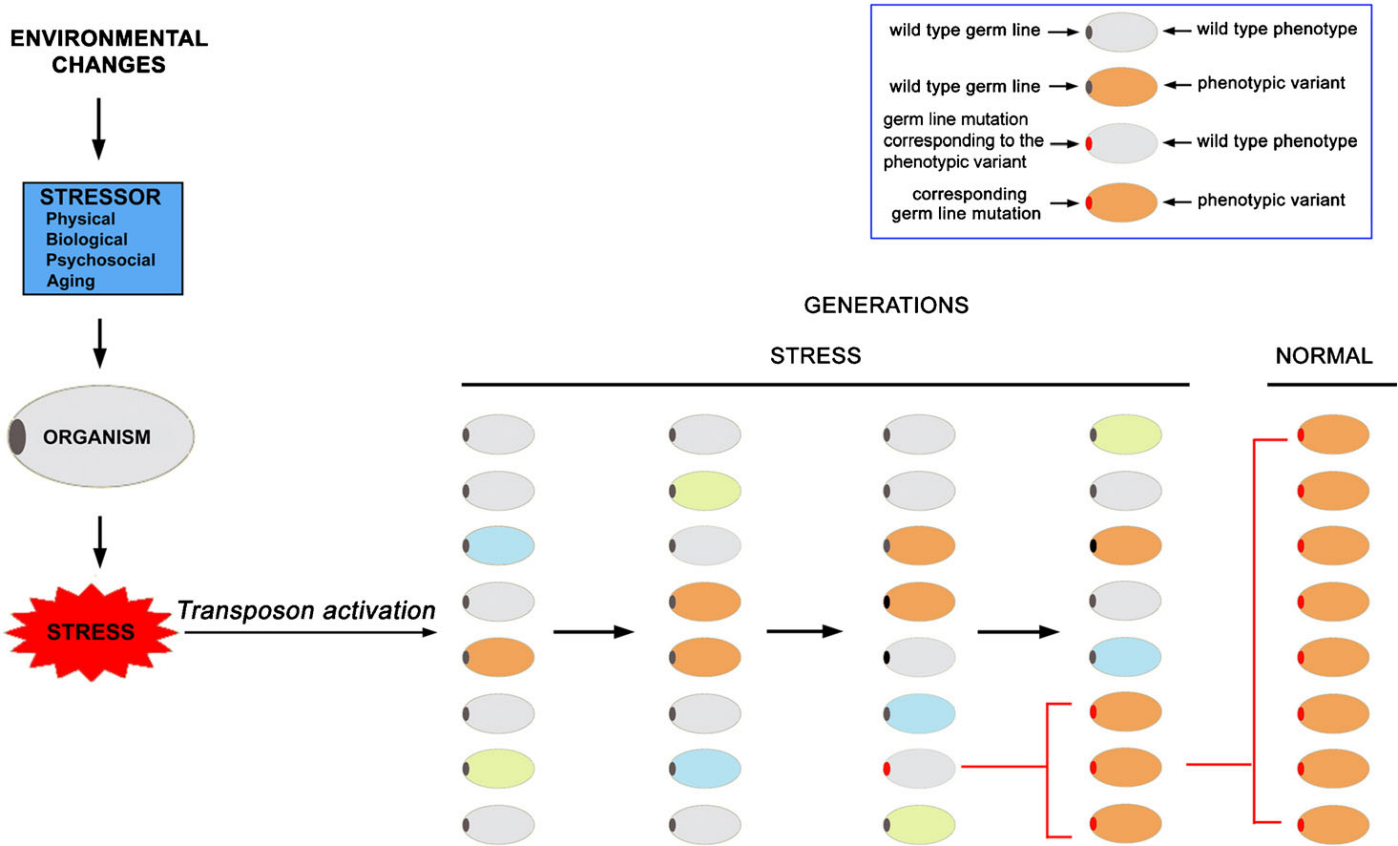
In nature, environmental changes can induce morphological variants and the activation of transposons due to the susceptibility of organisms to different types of stress that can be maintained through the generations. The activity of transposons could cause the insorgence of a germinal mutation corresponding to one of the phenocopies that could be then fixed

The stress-induced activation of transposons may provide an explanation for what Waddington called the assimilation process (Fig. 2). In nature, environmental changes can induce morphological variants due to the susceptibility of organisms to stress. The stress on an organism could be seen, as suggested by Hans Selye (1936), as a “nonspecific response of the body to any demand.” Several types of stressors are known, and unlike the temporary stress of experimental conditions, environmental changes can become stable and continue to produce their effects until adaptation takes place. From our current knowledge, we can imagine that a continuous environmental stress over time might induce and maintain several types of morphological variants, some of which could be potentially beneficial and could persist through generations. This multigenerational phenomenon could also be independent from the transmission of gene mutations through the germ line. This may be due to repeated stress at each generation (similar to that observed by Waddington in his experiments) or to the persistence of alterations in epigenetic mechanisms of the control of gene expression, as has already been demonstrated in some cases (Cavalli and Paro 1998; Tariq et al. 2009; Seong et al. 2011; Stern et al. 2012). Thus, in a new environment, advantageous phenotypes could be maintained through many generations and then fixed by the selection of de novo corresponding to germinal mutations arising by chance that take over for those phenotypes. We imagine these mutations being induced by transposon activity before the restoration of silencing that would take several generations—until the stress conditions are no longer perceived as such—and all the mechanisms will return to the basic activity. Intriguingly, a similar mechanism called “organic selection” was proposed by Baldwin in 1896 and is known as the “Baldwin effect” (see Crispo 2007 for a discussion). However, at that time, this type of adaptive mechanism was considered irrelevant to selection theory. It was very difficult to envisage what sort of mechanism could produce de novo mutations, some of them corresponding to the stress-induced phenotypes, and then fix them in relatively few generations as Waddington found. The possibility that stressful environmental changes could induce the activation of transposons gives a realistic framework for the possible existence of adaptation mechanisms like organic selection.

Support for this idea comes from the observation that more than 80 % of all recovered spontaneous mutations in natural populations of *Drosophila melanogaster* are due to transposon insertions (see Ashburner et al. 2005). It is particularly interesting that spontaneous mutations recovered at the homeotic genes were due to transposon insertions (Bender et al. 1983). This implies that transposons have made a significant contribution to the onset of morphological novelty. In this light, it is not unreasonable to assume that, among the repertoire of mechanisms of evolution, transposons play a significant role.

The existence of a transposon-based mechanism to generate variability is also suggested from old and recent observations in *Drosophila*, showing that inbreeding generates both morphological variability (see Lerner 1954 for discussion) and transposon activation (Di Franco et al. 1992; Ratner and Vasilyeva 1996). It is well known that the deterioration of populations subjected to inbreeding is a general phenomenon. The simplest interpretation is that inbreeding unmasks deleterious recessive mutations. However, as discussed extensively by Lerner in his book on “Genetic homeostasis” (1954), there are cases, such as in rats and mice, in which a certain degree of variability is maintained even after a consistent number of generations of inbreeding. Such cases have raised the question of whether inbreeding leads to the expected homozygosity as theoretically calculated. Many researchers have also observed that inbreeding depression can lead to an increase in phenotypic variability that has been assumed to be of environmental origin. Relevant to this argument, inbreeding has been shown to cause transposon mobilization in *Drosophila*, again suggesting their involvement in generating phenotypic variability (Di Franco et al. 1992). Taken together, these observations suggest that inbreeding is perceived as a stress, triggering the activation of transposons. This would restore a certain degree of variability and mitigate the danger of homozygosity. Written in this way, the argument may seem teleological; however, the mechanism can be explained considering that isogenization can make an organism homozygous for defective alleles at genes involved in transposon silencing; the consequence would be transposon activation.

In conclusion, it appears fully reasonable that transposon activation, in terms of transcription and transposition, could be a major reaction of genomes to genetic and environmental stresses, thus representing a powerful adaptive response.

## Environmental change can induce rapid evolution by increasing both genetic variation and selection choices using transposons as accelerators

“Species of different genera and classes have not changed at the same rate, or in the same degree.” “…the periods during which species have undergone modification, though long as measured in years, have probably been short in comparison with the periods during which they retain the same form.”

Charles Darwin

We believe that our model has a wider theoretical significance in evolutionary biology and that transposon biology may shed light on classic questions about the mechanisms of evolution, such as reaction norm, genetic load, rapid evolutionary changes, and the evolution of evolvability. We propose that transposable elements can be seen as both accelerators of the evolutionary process and mediators of the evolution of evolvability. Our view could be relevant for an explanation of the Dobzhansky’s paradox. Dobzhansky, in his book entitled “Genetics and the origin of species” (Third edition 1951), pointed out the existence of an apparent paradox from the studies on genetic variability in natural populations: The majority of genetic variants that are retained in heterozygous condition are mainly deleterious and then cannot be seen as useful elements for evolutionary processes. An intriguing point discussed by Dobzhansky was that the mutability seems to be under genetic control and that populations with high rate of mutability would have a lower adaptive value with respect to those with reduced mutability. The suggestion is that natural selection would favor genotype with low mutability although an accumulation of germinal mutations would be necessary for preservation of an evolutionary plasticity. According to him, the process of adaptation may be looked at a series of conflicts between the organism and its environment, and his conclusion was that “An ideal genotype would be capable of producing an optimal response to any environment. It appears, however, that no organism has evolved such a paradigm of adaptability.”

In our opinion, the biology of transposons offers a way to resolve this paradox in a Darwinian framework and suggests that organisms have evolved efficient responses to environmental change. Perhaps, the mechanisms for silencing transposons are the best example of how selection favors populations with low mutability. These mechanisms have been selected as a defense against transposons, whose activity would be dangerous for the survival of the cell and then of the organism. However, a drastic environmental change could impair these mechanisms, and this could trigger mutability, leading to an increase of genetic variability.

## Transposons appear to be the ideal tools for evolvability and rapid evolutionary change

**Fig. 3:**
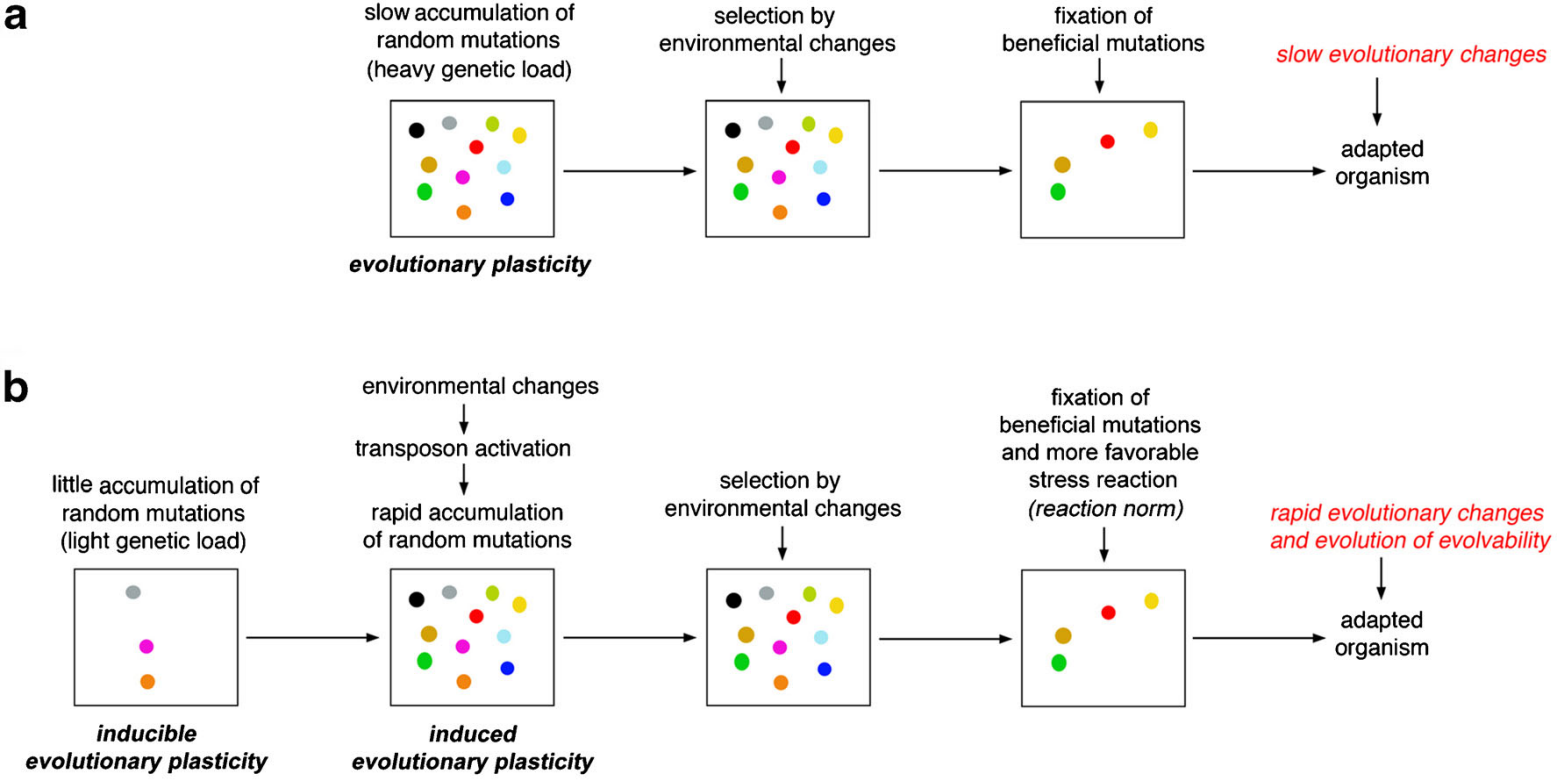
Two different views on evolutionary plasticity in populations. **a** Classical view on evolutionary change due to a slow and gradual accumulation of mutations, some of which could be selected by environmental stress. **b** It is possible to imagine a second view according to which there is a state of “inducible potential evolutionary plasticity” characterized by a limited variability. Under environmental stress, variability increases generating a new state of “induced evolutionary plasticity” on which selection could act establishing more favorable mutations. It is clear that from this point of view, the environmental stress may also select the strength of reaction norm

As illustrated in Fig. 3, we suggest a mechanism that would keep organisms in a state of limited variability but with an inducible evolutionary plasticity. This would ensure both a necessary basic genetic reservoir plus the possibility of a rapid increase in variability in the presence of environmental changes, thus generating a state of induced evolutionary plasticity. In other words, we can imagine the state of inducible evolutionary plasticity as the capacity to increase genetic variability by stress-induced transposon activity and the induced evolutionary plasticity as the actual increase of genetic variability by such a mechanism. Consequently, we call “pro-adaptation” as the ability to rapidly adapt to drastic environmental changes by an appropriate reaction norm which increases genetic variability and “pro-adaptors” as the genetic modulators of this reaction norm. Rather than a conflict between the genotype and the environment, we can hypothesize a sort of adaptive chain interaction, i.e., a process of “environmental action-genome reaction-natural selection-genome adaptation,” as discussed below. Although the evolvability is a concept that has been used with several different meanings and is still the object of controversial debates (see Brookfield 2001; Pigliucci 2008; Poole et al. 2003; Sniegowski and Murphy 2006 for discussions), by our view, evolvability could be considered as the property of a population to randomly react to environmental changes by increasing the variability among individuals and thus increasing the adaptive chances. This also makes the concept of adaptive mutations induced by environmental stresses unnecessary; it supports the classical principle of genetics that the probability of the occurrence of a mutation is independent of its effect on phenotype.

In conclusion, this view clearly challenges the idea of evolutionary change being merely due to a slow evolutionary genetic process: Considering the adapted species in a given environment, it is not necessary to invoke a slow and gradual accumulation of mutations with its concomitant risk of a heavy genetic load. Transposon silencing mechanisms leave populations dealing with a small accumulation of mutations. In the presence of environmental stress, this accumulation will be rapidly increased by transposon activation with the consequent rapid increase of variability for natural selection to act on. However, these two hypothetical modes of speciation, although theoretically different in their mechanisms and temporal dynamics, would be similar in their outcomes under the selective pressure of environmental stress. A transposon-based mechanism is not far from Darwin’s thinking who believed that the periods in which the species were subjected to modifications were shorter than those in which the species were persistently unchanged (Darwin, The origin of species, chapter XV, 1859). In other words, evolution is gradual but does not progress at a constant speed. This inconstant speed could be due, at least in part, to the activity of transposons that would act as evolutionary *accelerators* in the presence of environmental changes.

## Concerted role of transposons and chaperones during environmental stress

It is well known that genomes under stress, such as after a strong heat shock, undergo a substantial silencing, probably to avoid the potential danger of incorrect folding of proteins. However, since the functionality of some proteins is essential for survival, their correct folding must be insured by the action of induced chaperone complexes. The activation of chaperone complexes and transposon activity seems to fulfill a dual, concerted role. On one hand, the activated transposons increase the variability and play an adaptive role; on the other hand, the chaperone complexes modulate genome expression, thus playing a protective role. Intriguingly, transposon activation also seems to play a role in genome silencing during stress. For example, it has been shown that Alu RNA, transcribed from human SINEs, acts as transcriptional repressor in cells during heat shock (Mariner et al. 2008). Therefore, we postulate that in nature, the mechanism of transposon silencing protects individuals and, when inactivated by environmental stress, increases the evolutionary potential of populations; in other words, the induction of new individual variants produces an increase of variability inside the populations. Once again, this will confer a greater evolutionary plasticity.

The effect of Hsp90 on transposon activation suggests that the Hsp90-interacting chaperone Hsp70 (Mayer and Bukau 2005) with their modulator Hop (Johnson et al. 1998), whose functions can be induced or reinforced by different types of stress, could also directly induce transposon activity by repressing piRNA formation. In this case, Hsp70 would appear to be the main mediator in the stress response, playing a role in increasing both the survival protection of individuals and the genetic variability in their germ cells.

## Modes of induced plasticity by transposons

In general, evolutionary plasticity induced by transposon activity can be regarded as the reshaping of genomes by rearranging chromosomes and increasing the frequency of both structural gene and regulatory sequence mutations (Schmidt et al. 2012; see Shapiro 2010 for a discussion). Rearrangements and mutations in regulatory sequences are of particular importance because they change the modulation of gene expression and the regulatory networks in which they are involved (Lerman et al. 2003; Faulkner et al. 2009; Ellison and Bachtrog 2013; see also Feschotte 2008; Bourque 2009; Cowley and Oakey 2013 for reviews). The quantitative and qualitative changes in gene expression may be subtle: Instead of causing a deleterious mutant, they might create a modified gene interaction network with a strong adaptive potential. A striking example is the demonstration that a specific transposable element has contributed significantly to the evolution of pregnancy in mammals by mediating the rewiring of novel gene regulatory networks (Lynch et al. 2011). Another proposed evolutionary mechanism is a “transposon molecular domestication” consisting in the transition of a transposable element-derived coding sequence to a stable integrated gene that is beneficial to the host (Miller et al. 1992; see also Miller et al. 1999 and Pinsker et al. 2001 for reviews). One relevant example of molecular domestication seems to be the paired domain of the Pax6 protein, a conserved master regulatory gene of eye development. It has been proposed that this domain is derived from an ancestral transposase (Breitling and Gerber 2000). Perhaps, the notable increase in transposon transcripts under stress conditions also has important evolutionary implications. These transcripts could determine an adaptive rewiring of regulatory networks by inducing changes in the epigenome or by the modification of regulatory gene interactions guided, for example, by microRNAs. This latter possibility is truly intriguing, since it has been shown that many microRNAs have a transposon origin (Piriyapongsa et al. 2007; Piriyapongsa and Jordan 2008; Voinnet 2009). It is not unreasonable to assume that an increase in transposon transcripts, similar to what has been shown for competing endogenous RNA (ceRNA) (Salmena et al. 2011), may reduce the availability of microRNAs by a *sponge effect*, titrating these small RNAs and then changing the regulatory networks in which they are involved. If these modifications could be maintained for multiple generations, as have happened in some cases (Suter and Martin 2010; Ashe et al. 2012), it would be also possible that specific transposon-induced de novo mutations could take control of these newly formed regulatory networks and fix them as discussed above (Fig. 2).

## Transposons and the evolution of evolvability

“Selection deals not with the genotype as such, but with its dynamic properties, its ‘reaction norm,’ which is the sole criterion of fitness in the struggle for existence.”

Theodosius Dobzhansky

If during environmental stress, the genome is physiologically protected by slowing down its metabolism while its adaptive plasticity is increased by transposon activity, we would observe opposite effects in different environmental conditions: In normal conditions, transposons will be mainly repressed and genomes will be active; under stress conditions, transposons will be activated and genomes will be largely repressed. However, as discussed above, a general activation of transposons can be extremely dangerous, leading to lethality or to phenomena such as the hybrid dysgenesis as observed in *Drosophila*. Therefore, to avoid serious damage to the genomes, it appears necessary to invoke a modulation of the transposition activity of some mobile elements and perhaps the maintenance of the repressed state for others. The modulation of transposon activity under stress could be under selection: Genotypes with a strong transposition response should not survive and be negatively selected; genotypes that react with a mild transposition response should survive and be positively selected. In other words, it is the reaction norm of the strength of transposon activation that is under selection.

Summarizing what has been discussed and as illustrated in Fig. 3, we can envisage the molecular mechanisms that maintain a low genetic variation and how the genes underpinning those mechanisms might themselves adaptively evolve. We propose that environmental changes play a direct, active role in the evolution of genomes by inducing genetic variability, thus allowing the selection of more adapted genotypes along with their more adaptive stress response. The latter can be considered as an adaptive susceptibility or adaptive reaction norm, i.e., transposons may make evolvability evolvable!

## Conclusions and perspectives

“Evolution is not just history, is the rhapsodic present that contains the past and, although unpredictable, the future”

(The authors)

Environmental changes, in their ability to induce transposon activation, could be formally considered as both hypermutators and selectors at the same time, leading to an acceleration of the evolutionary process. On the other hand, transposons can be viewed as a trigger of the speciation process through the production of variability and reproductive isolation of new species. This suggests an effective process for rapid evolutionary change and makes it clear why transposons and host genomes, although biological antinomies, have coevolved mechanisms that regulate transposition and mutational outcomes under environmental stress and optimize reciprocal survival in normal environmental conditions. The defense mechanisms and their alterations allow mutual survival of host and mobile elements. Under normal conditions, the silencing of transposons allows hosts to remain undamaged and at the same time allows these elements to remain integrated in the genome. Under stress conditions, the activation of the transposon causes variability and generates individuals with a greater probability of survival, which also results in a greater survival of the transposons themselves. At the end of the process, however, both the host and mobile elements will have been modified. The balanced conflict between the host and transposons could metaphorically be viewed as a race car on the evolutionary track ready to shoot forward at the starting stress signal.

Recurrent dialectical interactions between biological antinomies that produce reciprocal changes could be the logic that governs evolutionary processes in a sort of dialectical evolution.

We want to emphasize that this view is not a philosophical view but refers to biological and physical mechanisms. Moreover, it does not imply any teleological content and fully adheres to the logic that evolution cannot see the future. Each mechanism or event we have discussed, although it may sometimes appear ad hoc, can be perfectly interpreted as the result of chance and natural selection and not the consequence of something that was planned.

Future studies will surely give us information about the relationship among different types of abiotic and biotic stress and transposon activity. Most importantly, the application of genome-wide technologies will tell us how significant has been the role of preexisting genetic variation versus de novo induced genetic variation by transposons and other mechanisms, such as RNAi or epigenetic modifications, in the phenotypic variation and speciation in groups of organisms that are suitable for these type of studies. Among them, we anticipate that lizards, whose extreme phenotypic plasticity is well known, seem to be particularly promising.

For examples, the green anole lizards represent one of the best examples of adaptive radiation. Their diversification into hundreds of species, through interspecific competition and natural selection, has been well documented across the Caribbean islands (Losos 2009). The genome sequence of *Anolis carolinensis* has in fact already suggested a strong evolutionary dynamism of transposons (Alföldi et al. 2011; Tollis and Boissinot 2011).

A similar picture also appears for the *Podarcis* lizards, whose diversification in a variety of small ecological niches along the Mediterranean coasts is well documented. Particularly striking, it has been recently shown that a population of *Podarcis sicula*, arising from few pairs of individuals brought into a small Croatian island 36 years earlier, evolved differences in head morphology, bite strength, and digestive tract despite the short time scale from their introduction into a novel environment (Herrel et al. 2008). An involvement of transposons in such process is going to be investigated.
